# Inter-row cropping and rootstock genotype selection in a UK cider orchard to combat apple replant disease

**DOI:** 10.1186/s42483-023-00184-y

**Published:** 2023-07-04

**Authors:** Chris Cook, Naresh Magan, Xiangming Xu

**Affiliations:** 1 NIAB, East Malling, Kent, UK; 2 Applied Mycology Group, Environment and AgriFood Theme, Cranfield University, Cranfield, Bedfordshire, UK; 3 Washington State University Tree Fruit Research Extension Center, WA 98801 Wenatchee, USA

**Keywords:** Apple replant disease, Rootstock soil rhizosphere, Microbiome

## Abstract

Apple rootstock genotypes confer different levels of tolerance to apple replant disease (ARD) and vigour to a newly replanted apple tree. A hybrid management system of rotating the rootstock genotype planted between successive generations and inter-row planting in the alleyways of orchards may minimise the severity of ARD symptoms. High-throughput sequencing of the fungal ITS and bacterial 16S rDNA regions was used to investigate the diversity, and differential taxa present in soils displaying symptoms of ARD. Candidate pathogens and beneficial microorganisms were correlated with the above-ground establishment of each rootstock genotype in a UK cider orchard. Our results suggest that the same rootstock or rootstock with closely related parentage to the previous rootstock had more severe ARD symptoms. Planting in the alleyway appeared an effective strategy to minimise the severity of symptoms irrespective of rootstock genotype. The planting location effect had a higher contribution to the variation in the rhizosphere microbiome than that of the rootstock genotype. No predicted causal agents for ARD could be identified to a taxonomic level to predict their function but two species associated with mycorrhizae, *Pteridiospora spinosispora* and *Paraglomus laccatum* were identified as inversely correlated with ARD severity and could be candidate beneficial species for apple, warranting further investigation and research. Our findings suggest that planting in the alleyways and planting rootstocks genetically dissimilar to the previously planted rootstock can be beneficial for tree establishment. We have also identified species inversely associated with ARD severity, making candidates for future research to test the antagonistic effect of the species against ARD pathogens in apple roots.

**Supplementary Information:**

The online version contains supplementary material available at 10.1186/s42483-023-00184-y.

## Background

Successive planting of apple trees in the same location can cause initially high-yielding orchard trees to have reduced establishment and lower yields. This can ultimately result in loss or removal of these trees (Mazzola and Manici 2012). This disorder has been termed Apple Replant Disease (ARD). ARD causes stunted growth, poor fruit appearance, root tip necrosis, reduction in root biomass, and a delay in initial fruit cropping by 2–3 years (Mazzola and Manici 2012; Liu et al. 2014; Zhu et al. 2014). ARD has historically been managed by chemical fumigation of the soils to remove any pathogenic causal agents present prior to replanting. However, many fumigants have since been banned due to their damaging environmental side-effects; although some of them such as Basamid^®^ are still shown to be effective at controlling ARD, the broad spectrum fumigants that remain are often less effective than their predecessors (Yim et al. 2017; Xu and Berrie 2018). Non-chemical management strategies including orchard management and rootstock selection have thus become paramount in ARD control.

Orchard management practices are also important to minimise ARD onset. Inter-row cropping, planting trees in the old alleyways between the previous tree rows, are alternative strategies previously shown to be effective to reduce the severity of ARD (Kelderer et al. 2012). This has been suggested to be mainly due to the presence of distinctly different rhizosphere microbiome communities between the tree rows and grass alleyways (Rumberger et al. 2004; Leinfelder and Merwin 2006; Deakin et al. 2018). Weed management is also important and must be included when replanting in alleyways due to the detrimental effect of weed competition on the establishment of young trees which could be even more severe than ARD in some cases (Xu and Berrie 2018; Deakin et al. 2019).

Rootstock selection is also a critical factor as they have differences in tolerance/resistance to ARD (Rumberger et al. 2004; Leinfelder and Merwin 2006; Fazio et al. 2012). Cider orchards tend to use semi-vigorous rootstocks, since these generally more vigorous rootstocks/varieties are less likely to be affected by ARD. These include MM106, which is more susceptible to ARD, and M116 which is more tolerant to ARD (Auvil et al. 2011; Wang and Mazzola 2018; Xu and Berrie 2018; Deakin et al. 2019). Some historically important dwarfing dessert orchard rootstocks can be very susceptible to ARD (Auvil et al. 2011). Geneva rootstocks (G16, G30, G41, and G210) have been shown to be more tolerant to ARD in some, although not all, affected soils, compared to Malling rootstocks (M7, M9, M26, and MM106) and have different bacterial rhizosphere species compositions (Rumberger et al. 2004; Leinfelder and Merwin 2006; Wang and Mazzola 2019). Replanting an orchard with a rootstock different to the previous rootstock genotype could be effective in reducing ARD but the genetics of ARD resistance in the rotated rootstock and its genetic relationship to the previous rootstock need to be taken into account when deciding which rootstock to choose for rotation (Xu and Berrie 2018; Deakin et al. 2019; Shuttleworth 2021). It is possible the relationship between the planting location and the genetic tolerance of the rootstock could produce an ARD suppressive rhizosphere microbiome, reducing the reliance on chemical fumigation of soils to remove ARD pathogens pre-planting.

In this study, we present a continuation of the work from Deakin et al. (2019) and report the results of tree growth and rhizosphere microbiomes in relation to rotating rootstock genotypes planted in both the previous tree station and the corresponding alleyway. The study aimed to assess (i) whether tree growth throughout the first five years after replanting is greater in the alleyway than in the corresponding tree station (hence ARD) to compare rootstock tolerance to ARD between genotypes; (ii) if the variability in rootstock tolerance to ARD corresponds with the previously planted rootstock genotypes, and (iii) could the differentiation in rhizosphere microbiomes between the trees in the alleyway and the corresponding original tree station contribute to relative effects of ARD?

## Results

### Above ground effect of rotating rootstock and planting in the alleyway

**Fig. 1:**
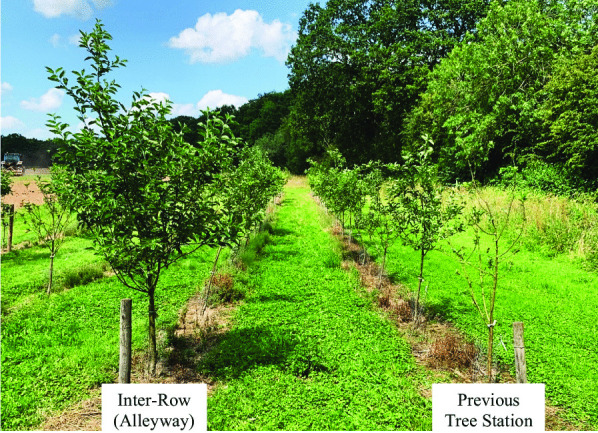
Above ground effect on MM106 rootstock genotype grafted to Worcester Pearmain scion. Both trees were planted in 2016 with the tree on the right planted in the previous tree station row and the tree on the left planted in the corresponding alleyway between the previous rows

Trees in the orchard established well in the early years of growth. All six trees grafted to G11 failed to establish, probably because of incompatibility with the Worcester Pearmain scion, or a latent viral infection within the scion (Ribinson et al. 2003; Deakin et al. 2019). Some tree pairs had a much healthier and more vigorous tree above ground in the alleyway compared to the corresponding tree station (Fig. 1). The more vigorous trees displayed increased branching, spread, height, and leaf area.

**Fig. 2:**
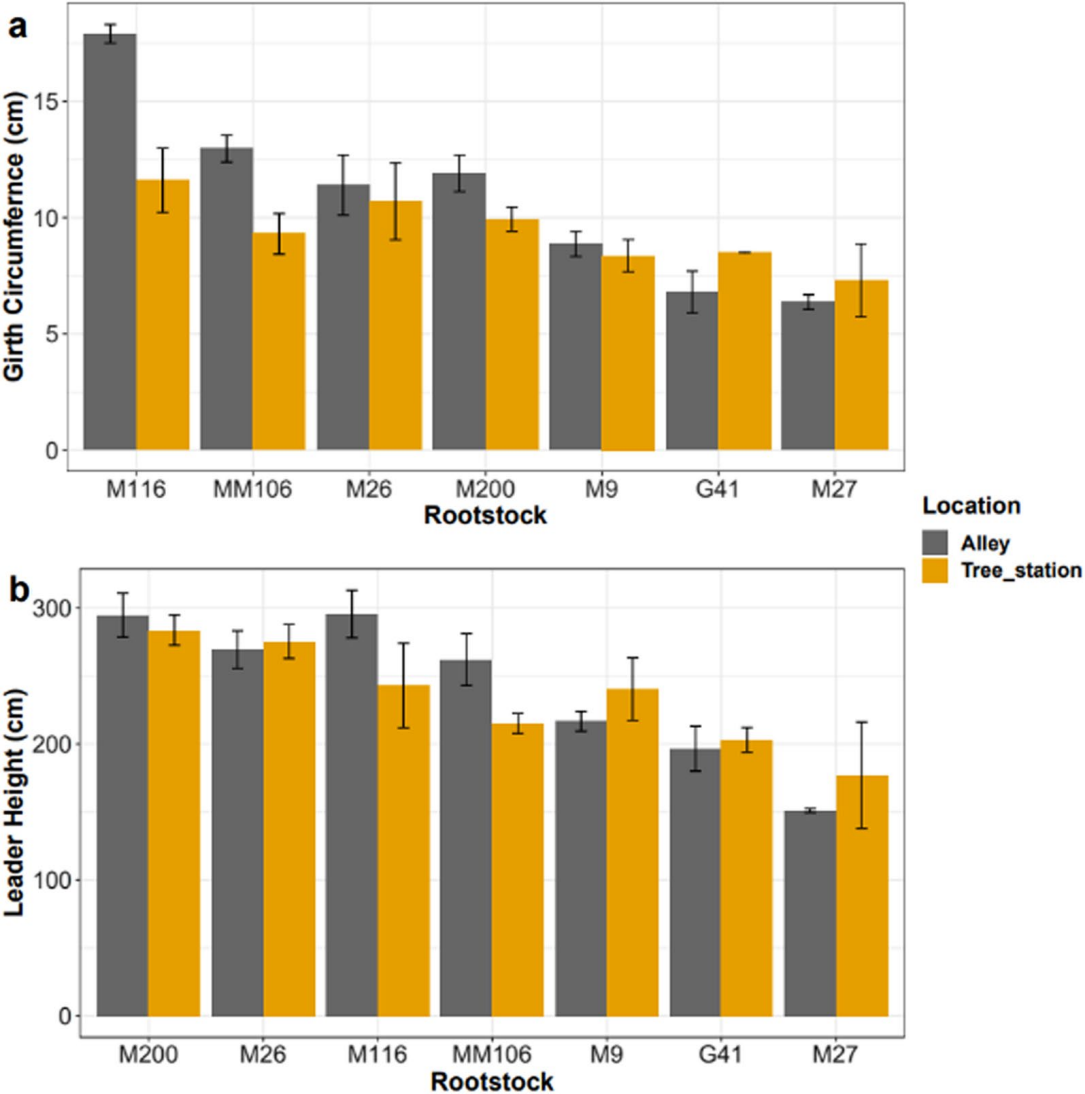
Mean girth circumference of tree trunks 5 cm above the graft union (**a**) and mean height of the leader from the graft union (**b**) in 2021. The colour of the bar indicates the location in which the tree was planted. Grey = Alleyway between previous rows, Orange = Previous tree station. n = 3, error bars are standard error

**Fig. 3:**
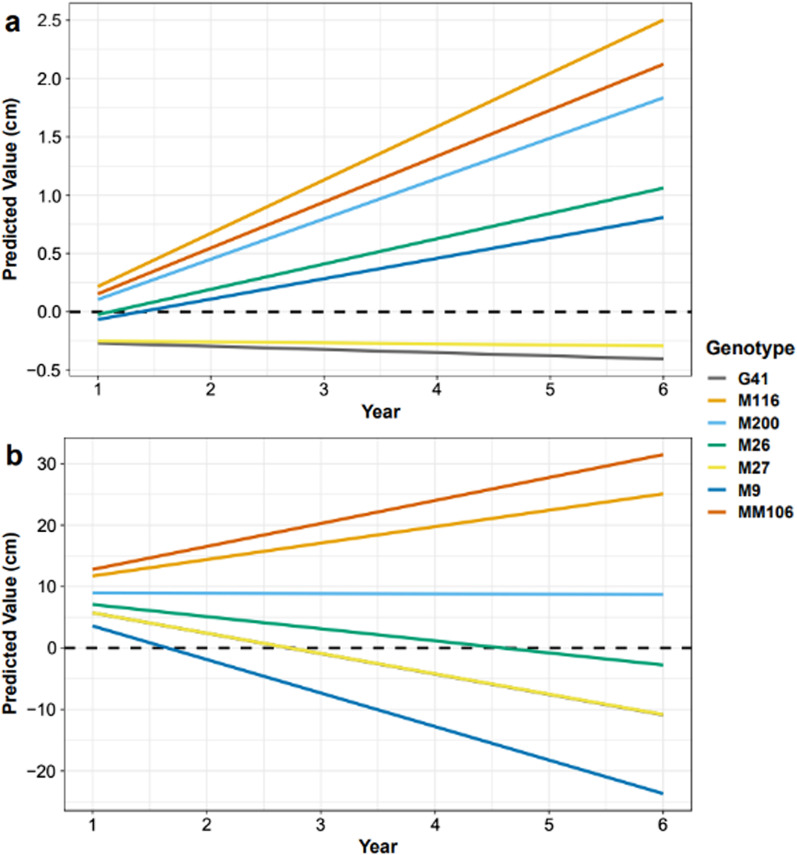
Predicted values from a randomly mixed model with a common intercept of temporal girth (**a**) and height (**b**) difference between the alleyway and the previous tree station based on measurements annually between 2016 and 2021. Genotype effect was fixed in the model and tree pair location treated as random. Slope trend was calculated from best linear unbiased prediction (BLUP) values. A value above zero indicates a replant effect on the tree planted in the tree station and a value equal to or below zero indicates no replant effect on that genotype

The difference (D) in trunk girth 5 cm above the graft union between trees in the alleyway and the tree station was negligible in 2016 (Table 1). In 2021, five of the seven rootstocks (M116, MM106, M200, M26, and M9) had a positive D value for the girth but only three (M116, MM106, and M200) of the five also had a positive D value for the height in 2021. The mean girth value for each rootstock genotype correlated well with the known vigour conferred by the rootstock to the scion and showed higher girth values in alleyway trees than those in the tree station in 2021 (Fig. 2a). The mean height value of each rootstock also followed the pattern of known vigour conferred to the rootstock (Fig. 2b). There was no clear pattern of better establishment in the alley as seen with the girth values in Fig. 2a with taller leaders in the alley (M116 and MM106), in the tree station (M9 and M27) or similar size between both locations (M26, M200, and G41). ANOVA analysis of log-transformed girth data across all time points showed a significant impact of rootstock genotype (*P* = 3e−4) and planting position (*P* = 0.001) on the girth of the trees and the interaction of genotype and planting position (*P* = 1.8e−5) (Additional file 1: Table S1). The slope estimate from the linear random mixed model was positive for the same five rootstocks estimate (Fig. 3a): the slope estimates were 1.36 (M116), 1.14 (MM106), 0.97 (M200), 0.52 (M26), and 0.37 (M9). The slope estimates for M27 and G41 were both less than zero: −0.27 and −0.34, respectively. From these results, an ARD score was assigned to each rootstock genotype: 0 for G41 and M27 (no ARD), 1 for M9 and M26 (intermediate ARD), and 2 for M116, MM106 and M200 (severe ARD). Similar analysis of the tree height (Fig. 3b) showed four of the seven rootstocks with a positive slope estimate: 22.13 (MM106), 18.41 (M116), 8.85 (M200), and 2.16 (M26). The remaining rootstocks had negative slope estimates: −2.54 (M27), −2.58 (G41), and −10.10 (M9).

**Table 1:** Average difference in girth and height between the alleyway and the previous tree station trees in the first year after planting (2016) and at the end of the trial (2021)

Genotype	Mean girth difference (cm)		Mean height difference (cm)	
	2016	2021	2016	2021
M116	−0.2 ± 0.16	5.0 ± 0.32	−14.8 ± 7.55	24.0 ± 3.27
MM106	0.03 ± 0.37	3.7 ± 1.19	2.8 ± 1.09	47.0 ± 16.82
M200	0.13 ± 0.24	2.0 ± 0.48	2.3 ± 10.04	11.0 ± 6.03
M26	0.2 ± 0.12	0.7 ± 0.46	12.0 ± 9.24	−6.0 ± 9.24
M9	0.1 ± 0.23	0.5 ± 1.2	8.5 ± 7.97	−23.7 ± 25.62
M27	−0.07 ± 0.12	−0.9 ± 1.79	4.0 ± 6.06	−26.0 ± 39.68
G41	0.1 ± 0.08	−1.7 ± 0.73	14.0 ± 4.90	−1.0 ± 23.68

The girth and height values for the tree in the tree station were subtracted from the values of the tree in the alleyway for each pair, and mean and standard error calculated. Hence, positive value indicated that the tree has greater establishment in the alley, negative has greater establishment in the previous tree station and 0 means that the trees are equal n = 3

Number after ± is the standard error value

The number of fruit on the trees in 2021 was low for all trees in the trial with the maximum number of fruit on one tree being 24 apples. In 17 of the 40 trees, there was no fruit when assessed in July 2021 before harvest. M116, M26, MM106, M200, and M27 all had higher mean fruit numbers in the alleyway than in the tree station (Additional file 2: Figure S1). M9 had a similar number of fruit in the alleyway and the tree station, whereas G41 had a higher number of fruit in the tree station.

### Summary of microbiome data

There was a total of 1,549,832 bacterial reads and 4,730,414 fungal reads across the 40 samples. The total number of OTUs generated was 10,883 for bacteria and 4802 for fungi. The number of reads per sample ranged from 25,305 to 45,970 for bacteria and from 2801 to 924,636 for fungi (Additional file 1: Table S2).

### Diversity indices

Four alpha diversity measures (Chao1, Shannon, Simpson, and InvSimpson) were calculated. Alpha diversity was generally higher for bacteria than for fungi. Bacterial alpha diversity was primarily influenced by the planting location with 15.5% and 26.5% of the variability in Shannon and Simpson/InvSimpson explained by the planting location, respectively (Table 2). Alpha diversity was higher in the original tree station than in the alleyway for bacteria (Additional file 2: Figure S2a). Rootstock genotype effect only accounted for 5.8% of the variation in Shannon indices, 2.3% for Simpson/InvSimpson, and 15.6% for Chao1. The interaction between genotype and location accounted for between 9% and 22.3% of the total variability but was not statistically significant. Most of the variability in the bacterial alpha diversity was unexplained by the experimental factors: 57.7% (Shannon, Simpson, and InvSimpson) and 59.8% (Chao1).

**Table 2:** Percentage of the variability in alpha diversity indices accounted for by block effect, planting location (alley vs tree station), rootstock genotype, and the interaction of planting location and rootstock genotype

Measure	Block		Location		Genotype		Location: genotype		Residual
	*P* value	%	*P* value	%	*P* value	%	*P* value	%	%
*Bacteria*									
Chao1	0.92	0.9	0.32	1.4	0.49	15.6	0.31	22.3	59.8
Shannon	0.49	5.0	0.02	15.5	0.84	5.8	0.44	16.0	57.7
Simpson	0.36	5.4	0.002	25.6	1.0	2.3	0.63	9.0	57.7
InvSimpson	0.41	5.4	0.003	25.6	1.0	2.3	0.70	9.0	57.7
*Fungi*									
Chao1	0.002	28.2	0.04	9.8	0.39	9.5	0.40	11.6	41.0
Shannon	< 2e−16	46.5	0.06	6.7	0.83	4.1	0.40	8.8	34.0
Simpson	< 2e−16	43.5	0.06	6.1	0.89	4.5	0.49	9.3	36.6
InvSimpson	< 2e−16	43.5	0.07	6.1	0.79	4.5	0.46	9.3	36.6

Alpha diversity for fungi was similar between the tree station and the alleyway, with the location accounting for between 6.1% and 9.8% of the total variability (Additional file 2: Figure S2b and Table 2). Both the genotype and the interaction between genotype and location effect were not statistically significant. There were large differences between blocks, contributing to 43.5% (Simpson/InvSimpson), 46.5% (Shannon), and 28.2% (Chao1) of the total variability. Fungal alpha diversity was lower for trees planted in the alleyway compared to those planted in the tree station within each of the ARD groups. Alpha diversity was similar between the three ARD groups for both bacteria and fungi (Additional file 2: Figure S3).

**Fig. 4:**
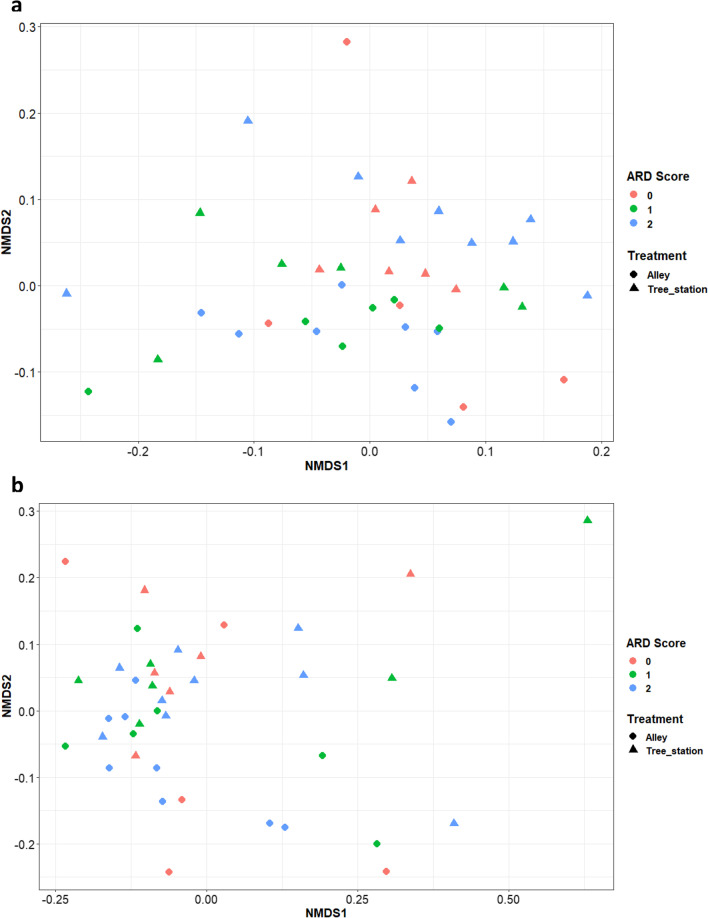
The first two dimensions of NMDS analysis of the Beta (β) diversity indices (Bray–Curtis dissimilarity) for bacteria (**a**) and fungi (**b**). Closer the distance between the points indicates more similarity in microbial communities between the samples. The Colour of the point indicates the ARD score associated with those rootstock Genotypes. The shape of the point indicates the planting position in the alleyway (●) or the previous tree station (▲)

Bray–Curtis beta diversity indices were used to represent differences in microbial communities in the rhizosphere among samples. The difference between the alleyway and previous tree station samples was most pronounced in the bacterial community than in the fungal community (Fig. 4). There were also differences in bacterial and fungal communities between ARD groups. ADONIS analysis highlighted the difference in beta diversity between blocks (*P* = 9.9e−4 for both bacteria and fungi) and between planting locations (*P* = 9.9e−4 for bacteria and *P* = 8e−3 for fungi) for both bacterial and fungal communities (Additional file 1: Table S3). The difference between ARD severity groups was statistically significant for the fungal community only (*P* = 0.03). There were significant interactions between the planting location and rootstock genotypes in the beta diversity indices for both bacterial and fungal communities, accounting for between 13.1% and 14.8% of the total variability (Table 3). The magnitude of the genotype and interaction effects did not change among the three sampling times, whilst the location effect slightly increased between T2 and T4 (Table 3).

**Table 3:** Percentage of variation in the rhizosphere microbiome accounted for by rootstock genotypes and within-orchard location (aisle or tree stations), and their interactions, as determined by analysis of variance of all principal component scores for each individual sampling points (T = Time (Year))

Measure	T0	T1	T2	T4
*Bacteria*				
Location	5.64	2.72	2.80	4.92
Genotypes		14.39	14.77	14.11
Location: genotype		14.41	13.96	13.11
*Fungi*				
Location	9.35	2.82	2.99	4.87
Genotypes		14.43	14.65	14.15
Location: genotype		14.38	14.33	13.07

### Differential OTUs between ARD and non-ARD trees

OTUs were compared between the trees with the most severe ARD (score 2) and the trees that did not show ARD (score 0). After DESeq2 filtering, approximately 80% of the representative OTUs for both bacteria and fungi were retained for comparison. There were no bacterial OTUs identified to have a differential abundance between the two ARD groups. Only a small number of fungal OTUs, 0.079% higher and 0.21% lower, significantly differed (*P* < 0.1) in their relative abundance. There was a high percentage (87%) of fungal OTUs with low counts filtered out by DESeq2 filtering but 0 bacterial OTUs with low counts.

Table 4 shows fungal OTUs with differential abundance from DESeq2 analysis, indicating putative ARD pathogens and beneficial fungi, and their associated SINTAX taxonomy predictions (at a confidence threshold of 0.65) and BLAST taxonomy. One of the OTUs predicted as a putative causal agent was identified as *Russula praetervisa*, a saprophyte. The remaining OTUs predicted as putative causal agents were not able to be assigned to a taxonomic rank low enough to predict ecology. Similarly, four of the eight OTUs identified as beneficial fungi were unable to be assigned with a taxonomy at a level sufficient to identify their potential function. The saprophytic genus *Podospora* was identified as more abundant in non-ARD trees. *Arthrinium arundinis* was more abundant in non-ARD trees and has been described as a plant pathogen of barley causing kernel blight but has also been described as a saprophyte and endophyte (Cano 1992). It is unlikely to be pathogenic in this study as its relative abundance was positively associated with healthier non-ARD trees. Two of the putative beneficial OTUs were associated with mycorrhizae *Pteridiospora spinosispora* and *Paraglomus laccatum* (Filer and Toole 1966; Renker et al. 2007).

**Table 4:** Differential OTUs from DESeq2 analysis to identify candidate causal agents for ARD and beneficial microorganisms working against ARD through the following comparison: (ARD rootstocks at Tree Station + Non-ARD rootstocks in Alleyway)—(Non-ARD rootstocks at Tree Station + ARD rootstocks in Alleyway) [namely the interaction between ARD genotypes and position]

DESeq2 model	SINTAX species/taxa*	BLAST taxonomy	Ecology	Base mean	LFC	*P* value
*Fungal candidate causal agents*						
	*Russula* (g)	R*ussula praetervisa*	Saprophyte	2012.9	0.033	0.026
	*Sebacinales* (o)	Uncultured *Sebacina* isolate	Unknown	121.1	1.669	0.033
	Unknown Fungi (k)	Unknown Fungi	Unknown	43.9	0.041	0.065
*Beneficial fungal candidates*						
	*Pleosporales* (o)	*Pteridiospora spinosispora*	Isolated from mycorrhizae of sweetgum	4997.6	− 0.129	0.026
	*Agaricomycetes* (c)	Uncultured *Agaricaceae*	Unknown	1581.6	− 0.069	0.094
	Unknown Fungi (k)	Unknown Fungi	Unknown	48.7	− 1.492	0.018
	Unknown Fungi (k)	Unknown Fungi	Unknown	67.0	− 0.114	0.018
	*Paraglomerales* (o)	*Paraglomus laccatum*	Mycorrhizae	33.9	− 0.073	0.065
	*Ascomycota* (p)	Uncultured *Podospora*	Saprophyte	51.2	− 0.098	0.094
	*Apiosporaceae* (f)	*Arthrinium arundinis*	Saprophyte/Endophyte/Plant pathogen of barley	122.6	− 1.502	0.026
	*Hypocreales* (o)	Uncultured *Hypocreales*	Unknown	87.3	− 0.126	0.086

Positive Log_2_ Fold Change (LFC) indicates higher OTU abundance in the first condition/treatment and vice versa for negative values. *P* values are Benjamini and Hochberg corrected for false discovery

*The lowest assignable taxonomic rank with a SINTAX confidence ≥ 0.65

## Discussion

This study has shown that different rootstock genotypes confer different levels of tolerance to ARD in a UK cider orchard. We also noted that ARD severity was most severe in rootstocks closely related to the previously planted rootstock and suggest that there may be a genetic link factor between successive generations of rootstocks. However, future studies would be required to confidently draw this conclusion. Better tree establishment was observed for those rootstocks suffering from ARD in the alleyway than in the previous tree station. Both alleyway and original tree stations had distinct bacterial and fungal communities in the rhizosphere; such differences appear to become stable within the first year of replanting. All fungal OTUs predicted as candidate ARD causal agents could not be effectively assigned to taxonomic ranks which were sufficiently low enough to predict pathogenic effects in apples, or those that were previously reported as saprophytic. Of the eight fungal OTUs identified as potential beneficial microorganisms, two were reported from mycorrhizae and another (*Arthrinium arundinis*) had antifungal and cytotoxic compounds (Zhang et al. 2018) making it an amendment candidate for a role in biocontrol.

Each rootstock confers different levels of vigour to the scion, so it sometimes becomes difficult to assess the extent of ARD between rootstocks. For instance, a vigorous rootstock can still confer better growth under ARD conditions to scions than dwarfing rootstocks that do not suffer from ARD. In this study, we measured the severity of ARD as the relative difference in tree establishment between pairs of the same scion grafted to the same rootstock genotype planted in the previous tree station and the corresponding alleyway. The present results suggest that five of the seven tested rootstocks showed a varying degree of ARD, with MM106, M116, and M200 experiencing the most severe ARD. MM106, M116 and M200 are not known for their susceptibility to ARD as they are all vigorous rootstocks. Thus even when suffering from severe ARD (planted in the original tree station), these rootstocks still conferred better tree growth than M9, G41 and M27. Only by comparing tree development between the alleyway and the previous tree stations does the ARD effect become apparent for these vigorous rootstock genotypes. This finding also suggests another possible way of combating ARD—planting more vigorous rootstocks in previous tree stations if no other methods of managing ARD are economically viable. Further studies on fruit size and quality would also be necessary to confirm that tree growth aligns with fruit quality.

The above-ground effect on the most severely affected trees was consistent with the symptoms of ARD, showing stunted growth, reduced vigour and discolouration of leaves (Mazzola and Manici 2012). The present result suggested that ARD is not limited to the first few years after planting but can persist beyond five years after planting—the difference in the tree development between the alleyway and tree station increased with time at a constant rate as indicated by the constant slope. The previously planted rootstock in the orchard was MM106; M116 is derived from the cross between MM106 and M27, implying a possible genetic link between the newly planted rootstock with the previous rootstock in terms of susceptibility to ARD. The present results provide preliminary evidence that planting a genetically different rootstock from the previous rootstock could be effective to reduce ARD development. As the main focus of this study was looking at tolerance differences between rootstock genotypes, we propose additional work looking at large-scale field rootstock genotype succession as the main focus of the study. Interestingly, M27 did not exhibit any ARD effects in the late years despite being identified as susceptible to ARD in year 2 (Deakin et al. 2019), highlighting the unpredictable nature of ARD during the establishment of young apple trees.

Girth differences are a better measure of the effect of ARD than height differences in this particular study. Many external factors including mechanical damage of leader branches, pruning of leader branches to remove canker lesions or high wind damage could all impact the tree height, independent of experimental factors. However, these factors would not be expected to affect tree girth directly. This could explain why the height data were not synchronous with the girth data in this particular study. Similarly, yield data were highly variable and low across all rootstocks in the trial. In general, the more vigorous trees in the pair would have a higher fruit number due to more branches being able to bear fruit. The low fruit number could be due to the early removal of fruit by external influences or issues during the blossom period although there were no significant differences in blossom timing.

Temporally, the differences in rhizosphere microbial communities between the two locations (the previous tree station and the corresponding grassy alleyway) would be expected to be reduced due to the recruitment of similar microbes from the bulk soil by the same rootstocks (Deakin et al. 2019). However, this was not supported by the present results. The contribution of the planting location to the total variation in microbial communities remained significant and had not changed much after five years of establishment. One possible explanation for this observation could be the functional redundancies in the bulk soil microbial communities. Different rootstock genotypes may recruit microbes to the rhizosphere with similar functions, but the exact composition of microbes with specific functions may be different in the alleyway and the previous tree station due to long-term effects of herbicide applications, previous vegetation and soil compaction.

Additionally, the interaction between the rootstock genotype and the location was a significant contributor to the overall variation in bacterial alpha diversity. These results suggest that although the plant host plays an important role in shaping the rhizosphere associated with their roots through root exudation (Haichar et al. 2008; Burns et al. 2015; Leisso et al. 2017; Guyonnet et al. 2018), the differences between the tree station and the alleyway bacterial communities could lead to functional redundancy and may partially explain the disparity between bacterial populations between the two locations after seven months (Deakin et al. 2019). The effect of genotype remained stable throughout the study suggesting that the microbial recruitment from bulk soils by rootstock genotypes is relatively rapid. This highlights the importance of the early application of soil amendments especially of beneficial microorganisms to maximise recruitment into the rhizosphere of the young tree and improve the early tree establishment.

The blocking effect significantly influenced the fungal diversity in this trial implying the position within the orchard was more influential than the planting position or the genotype planted. The trial was carried out at the bottom of an orchard on a slope leading to a small stream at the base. The proximity of some blocks to the stream or water movement downwards along the slope through the blocks may have altered the soil moisture content across the orchard. This in turn would have influenced both the dominant microbiota more suited to higher moisture conditions and the soil physio-chemical properties such as pH and soil texture that all highly influence the soil microbiome (Fierer 2017). The differences between ARD groups were only significant for fungi, suggesting that this group are more important for ARD onset in the study area. This is consistent with the role of ARD pathogens which are predominantly fungal or oomycetes with no bacteria definitively identified as causal agents (Somera and Mazzola 2022).

Using the beta diversity analyses for the communities of bacteria and fungi present in both the tree station and the alleyway showed consistent differences in the communities present between the two locations for both microbial groups. This was similar to what was observed in the alpha diversity analyses. The difference in communities was probably due to factors such as herbicide application, microbial recruitment through exudation from the tree roots and the alleyway having previous vegetation combined with compaction via the use of heavy machinery. There was also a difference in the communities for both bacteria and fungi between rootstocks planted in the two areas within each ARD score group. In particular, the rootstocks with severe ARD had clear differences in the communities in the rhizosphere of trees in the alley and the tree station. Functional redundancy again could be a factor contributing to this effect, as the overall difference in the community was different between the alleyway and tree station with the trees perhaps recruiting different microbes, but with similar functions. These results, therefore, suggest that differences at a community level in the rhizosphere alone are not directly correlated with ARD severity. This study only looked at the communities in the rhizosphere compartment but investigation of communities in the endophytic root-colonising compartment such as the work by Kelderer et al. (2012) would provide a more accurate identification of the causal agents of ARD in future studies.

The study identified three fungal OTUs as potential causal agents of ARD and eight fungal reads as potential beneficials. However, no bacterial OTUs were found to be associated with ARD. No bacterial genera were identified as causal agents or beneficial in this study. This is similar to what was found previously that bacteria were not the causal agent of ARD (Mazzola and Manici 2012). In addition, it is interesting to note that beneficial bacteria as biocontrol agents or as PGPRs did not increase in abundance and appeared to have little effect on ARD. This is in contrast to studies that have shown beneficial effects of a number of bacterial strains on ARD including *Bacillus* spp. and *Pseudomonas* spp. (Utkhede and Smith 1992; Sharma et al. 2017; Duan et al. 2022). Of the three fungal OTUs predicted as causal agents, only one OTU was identified to the species level as *Russula praetervisa*, a saprophyte in soil with no known pathogenic implications in apple. This highlights the difficulties in identifying causal agents for ARD by the use of sequencing. Although there may be relative differences in the abundance of OTUs, it does not guarantee that the OTU will be causing disease. Similarly, pathogenicity is rarely as simple as increased abundance equates to disease symptoms. However, a small increase in pathogens or functional changes in pathogens may be sufficient enough to result in phenotypic symptoms of ARD to become evident. Members of the oomycetes and nematodes are also important causal or exacerbating agents of ARD (Tewoldemedhin et al. 2011) that were not specifically profiled in the present study. Oomycetes can be detected using the ITS primers but were not of a high enough quality to accurately identify oomycete pathogens linked to ARD. No pathogenic oomycetes were detected with the selected primers, so future studies may need to focus on bacterial, fungal, oomycete- and nematode-specific primers for sequencing alongside a quantitative assessment of known ARD pathogens with specific primers to identify candidate causal agents from each community.

Two of the fungal OTUs identified as beneficial against ARD were likely to be *Pteridiospora spinosispora* and *Paraglomus laccatum*, two species of mycorrhizae (Filer and Toole 1966; Renker et al. 2007). Mycorrhizal inoculations of apple seedlings have been shown to suppress ARD symptoms and aid the establishment of the trees but not effective against oomycete pathogens (Čatská 1994; Xu and Berrie 2018). Increased abundance in non-ARD trees may suggest a reduced abundance of mycorrhizae in the tree station available for root colonisation. Amendments with mycorrhizae identified in this study, or similar commercially available species, could be a viable strategy for future studies to reduce/minimise or prevent ARD in replanted trees.

One fungal OTU identified as beneficial was the species *Arthrinium arundinis*. Previously, *A. arundinis* was described as a pathogen causing kernel blight in barley and leaf edge spot of peach, despite being correlated with non-ARD trees in the present study (Cano 1992; Ji et al. 2020). *A. arundinis* has also been shown to produce antifungal and cytotoxic compounds when isolated from the leaves of tobacco (Zhang et al. 2018). Thus, it is possible that the production of such compounds could function as components of the biocontrol strategy of this species which contributes to competition with the causal fungal agents of ARD. This study suggests that further investigation of the culturing and application of *A. arundinis* as a biocontrol agent for the control of ARD would be useful.

## Conclusion

ARD is a complex disorder with prevention heavily reliant on broad-spectrum chemical fumigation. This study sheds light on non-chemical management strategies for ARD utilising planting position and rootstock genotype selection. This work adds to the research to move towards non-chemical management of ARD, particularly in organic systems globally. With an integrated approach of physical in-field strategies highlighted in this study and biological pre-plant amendment of soils in recent studies, there is the confidence of developing a non-chemical management of ARD in the near future.

## Methods

Experimental details on the orchard history, crop management, soil characteristics and experimental design for the study were provided previously (Deakin et al. 2019). Here we provide a brief description of relevant experimental details.

### Orchard design

The study was conducted on a cider orchard in the West Midlands of England in Worcestershire (latitude 52.251020, longitude −2.301711). Both rotating rootstock genotypes and planting position (alleyway vs tree station) were investigated. The study consisted of eight rootstock genotypes planted in pairs: in the previous tree station and the corresponding middle alleyway position approximately 2 m away from the tree station. Three randomised blocks were used to remove positional effects within the orchard, i.e. proximity to the stream downhill or proximity to the tree line to the East of the orchard rows. Each pair location within each block was randomly assigned to one of the eight rootstock genotypes. Before grubbing in 2014, the orchard had been ‘Katy’ apples on MM106 MM111 with the area of the orchard used in the study previously MM106 for 12 years (Deakin et al. 2019).

### Rootstock and scion selection

Eight rootstocks were selected for the study based on their tolerance to ARD, vigour and importance to the industry. The rootstocks used were M9 (unknown pedigree), M26 (M16 × M9), M27 (M13 × M9), MM106 (Northern Spy × M1), M116 (MM106 × M27), G11 (M26 × *M. robusta* 5), G41 (M27 × *M. robusta* 5), and M200 (*M. robusta* 5 × Ottawa 3) from the East Malling breeding programme. M27, G41, G11, M9, and M200 are all dwarfing rootstocks. M26 is a semi-dwarfing rootstock and M116 and MM106 are semi-vigorous rootstocks. M27, G41, and M116 are reported to be tolerant to ARD and M9 and M26 are the most susceptible to ARD out of the eight genotypes (Deakin et al. 2019). The root ball of each rootstock was washed before whip and tongue grafting to the cultivar ‘Worcester Pearmain’ in 2015. The trees were potted in a peat and sand mix and grown for 7 months to ensure that graft was taken. The land was sub-soiled and rotavated before planting to prevent compaction. Pairs of trees with similar girth/height of the same rootstock were planted for a given position pair: the original tree station and corresponding alleyway, in October 2015. Trees were managed the same as the rest of the cider trees on the site during the trial period. Weed control was used to prevent vegetation competition identified by Deakin et al. (2019).

### Growth measurements and statistical analysis

Deakin et al. (2019) tested only looked at microbiome community analysis, whereas this continuation study also looked at above ground effect on tree establishment due to planting position and rootstock selection. Initial measurements of height (from ground level) and girth (the circumference of the tree trunk 5 cm above the graft union) were taken for each tree. Each winter during dormancy (between January and March) from 2017 to 2021 trees were assessed for height of the leader (which was not pruned) and girth. Trees were marked at the point they were measured for consistency in girth measurements between time points. Height was measured from ground level to the end of the leader of the tree (not including any leaf height added to the branch at the leader’s tip). The yield was the number of fruit per tree due to an abnormally low number of fruit per tree (≥ 24 individual fruit, many with 0 fruit).

All statistical analyses were conducted in R V4.0.2 (R Core Development Team 2008). In the case where one of the pairs of trees had died (either alley or tree station) the corresponding healthy tree in the pair would be removed from statistical analysis. The difference (D) in both height and girth was calculated between the alleyway and tree station for each tree pair and used in subsequent statistical analysis.

A simple ANOVA with Block, Year, planting position in alley or tree station (Position), and rootstock genotype all set as fixed variables and tree pair (position of the tree pair within the orchard) set as a random variable was used to see the effect of planting position and genotype on trunk girth data. Girth data was normalised using a log transformation prior to ANOVA analysis.

A linear mixed model with a common intercept was used to model the planting position effect on the D over time using the lme4 package v 1.1-28 (Bates et al. 2015). As described in Deakin et al. (2019), it is inappropriate to compare tree growth directly among genotypes so rather than relative rate of increase this study utilised a linear mixed model. The genotype variable is fixed in the model whereas the location of the tree pair D is calculated from is treated as a random variable. The package ggeffects v1.1.1 (Lüdecke 2018) was used to calculate predicted best linear unbiased predictions (BLUP) for the slopes in the model and visualised using ggplot2 package v3.3.5 (Wickham 2011). The slope estimate represented the extent of ARD; positive slope = ARD (trees in the alleyway grew faster than in the original tree station), and ≤ 0 = no ARD. Based on the slope estimates, an ARD score was assigned to each rootstock genotype: 0 (no ARD), 1 (intermediate ARD), and 2 (severe ARD) as the rootstocks separated into three separate groups for girth comparison measurements. Fruit yield was calculated as the mean number of fruit per tree was calculated and visualised in ggplot2.

### Sampling rhizosphere soil

This study looked at the rhizosphere soil associated with apple roots rather than bulk soil cores investigated in the previous study by Deakin et al. (2019). Rhizosphere soil was collected from each tree by using a sterile trowel or fork to dig under the tree to an approximately 10 cm depth and detach the roots from the tree. Soil that was attached to the root after light shaking was classed as rhizosphere soil. This soil was removed from the root into a polythene sample bag and immediately cooled in an electric cool box. There were a total of 48 samples collected on the site. Between samples, tools, and gloves were cleaned with 70% ethanol to prevent mixing of samples. Samples were transported to NIAB, East Malling, Kent, UK at 4°C and subsequently stored at 4°C for 24 h until molecular processing.

### Amplicon-sequencing of the rhizosphere soils for bacterial and fungal communities

#### Soil DNA extraction

Amplicon sequencing and sequence processing followed the method described previously (Deakin et al. 2018). In summary, genomic DNA was extracted from a 0.25 g subsample of the rhizosphere samples using the DNeasy PowerSoil Kit (Qiagen, Carlsbad, USA) along with a bead-beating benchtop homogenizer (Fastprep FP120, Qbiogene, Carlsbad, USA). DNA concentration and quality were determined using a spectrophotometer (Nanodrop 1000, Thermo Fisher Scientific, Cambridge, UK) and a fluorometer (Qubit 2.0, Thermo Fisher Scientific, Cambridge, UK). Twenty µl of DNA sample was sent to Novogene (Cambridge, Cambridgeshire, UK) for library preparation and amplicon sequencing.

#### Amplicon sequencing

The quality of DNA samples was first checked. PCR amplification was then performed for bacteria using the 16S V4 region amplified with the primer pair Bakt_341F/Bakt_805R (Herlemann et al. 2011). For fungi, the ITS1 and ITS2 regions were amplified with the primer pair EkITS1F/Ek28R (≡ 3126 T) (Gardes and Bruns 1993; Sequerra et al. 1997). PCR product was purified before library preparation. Sequencing was performed using the Illumina NovaSeq 6000 platform. Reads were paired-end 150 bp in length.

#### Sequence read processing

Sequence data were submitted to the NCBI database (Project PRJEB52534). FASTQ reads were demultiplexed. Ambiguous reads that did not match the forward and reverse read primers for 16S and ITS were removed before further processing. All analyses were conducted using USEARCH 11.0 (Edgar 2013) unless otherwise specified.

Bacterial and fungal reads were processed separately to create separate representative operational taxonomic units (OTUs) for bacteria and fungi. ITS forward and reverse reads were aligned with a 10% threshold of maximum difference in overlap and 16S reads were aligned with a 5% threshold. Primer sequences for forward and reverse reads were removed at this stage. Merged reads with adaptor contamination or total length fewer than 150 nucleotides for ITS reads or fewer than 300 nucleotides for 16S reads were removed. Quality filtering of the merged reads was conducted using a maximum expected error threshold of 0.5.

#### OTU generation

Unique sequences were identified and any unique sequence with fewer than 4 reads were discarded for OTU generation. Sequences were sorted by order of decreasing read numbers; OTUs were generated by clustering the unique sequences at 97% sequence similarity and a representative sequence for each OTU was also produced. Then all sequence reads that passed initial quality filtering were mapped against the OTU representative sequences to generate the OTU counts tables for ITS and 16S. To predict the taxonomy of the OTUs, a SINTAX algorithm (https://www.drive5.com/usearch/manual/sintax_algo.html) was used by aligning ITS OTU representative sequences to the reference database ‘UNITE v8.3’ (Nilsson et al. 2019) and 16S OTU representative sequences to the RDP training set v18 (Cole et al. 2014).

### Statistical analysis of sequencing data

Rarefaction curves were produced in the vegan R package v2.5.7 (Dixon 2003) to identify if samples were adequately sequenced. OTUs with the total number of reads fewer than 3 were discarded from further analysis. The vegan package was used to normalise the OTU counts data by rarefaction before further analysis.

Both alpha and beta diversity indices were calculated for fungi and bacteria continuing from the diversity measures produced by Deakin et al. (2019). Alpha (α) diversity indices (Chao1, Shannon, Simpson, and invSimpson) were calculated within the Phyloseq package v1.34 (McMurdie and Holmes, 2013) from the rarefied counts. Permutation ANOVA analysis in the LmPerm package v2.1 (Wheeler 2016) was used to assess the effects of planting location (alleyway vs tree station) and rootstock genotypes on alpha diversity. The rootstock genotypes were further divided into three groups based on the ARD score. The R package ggplot2 was used to visualise the alpha indices produced by Phyloseq. Beta (β) diversity index (Bray–Curtis) was calculated in the vegan package from the rarefied counts data. To visualise dissimilarity between samples, non-metric multidimensional scaling (NMDS) plots were used to visualise the differences in the beta diversity. Permutations of multivariate analysis of variance using F-tests based on the sequential sum of squares (ADONIS) was used to assess the effects of planting location and rootstock genotypes on beta diversity indices. Statistical significance was based on 1000 permutations.

The DESeq2 package v1.30.1 (Love et al. 2014) was used to identify OTUs with significant differences in the relative abundance between rootstock genotypes with the most severe ARD score and no ARD. Log_2_ fold change (LFC) was shrunk within DESeq2 when extracting results from the model with a *P*-value threshold of 0.1. The following specific comparison was used in DESeq2 analysis to identify candidate causal agents of ARD and beneficial microorganisms:

*(ARD rootstocks at Tree Station—Non ARD rootstocks in Alleyway) versus (Non ARD rootstocks Tree Station—ARD rootstocks in Alleyway)*

More abundant OTUs in this comparison were candidate causal agents for ARD and less abundant OTUs are candidate beneficial microorganisms associated with reduced ARD. Those OTUs identified with differential abundance identified by DESeq2 had their representative sequences run through NCBI’s Nucleotide BLAST search tool (https://blast.ncbi.nlm.nih.gov/Blast.cgi) to identify possible taxonomy since many OTUs cannot be identified to the level of genus or species with sufficient confidence via the SINTAX algorithm.

### Supplementary Information

**Additional file 1: Table S1.** ANOVA of log transformed girth data for planting position in alley or tree station and rootstock genotype. Block, Genotype, Year, and Position were set as fixed effects, whereas the tree pairwas treated as a random effect. **Table S2.** Summary of microbiome sequencing data. **Table S3.** Permutational multivariate analysis of variancebased on 1000 permutations of Bray–Curtis beta diversity accounted for by Block, Location, rootstock Genotype, ARD severity score, and interaction terms.

**Additional file 2: Figure S1.** Mean number of fruit counted on each tree in 2021. The shape of the point indicates the location in which the tree was planted. ● = Alleyway between previous rows, ▲ = Previous tree station. **Figure S2.** Alphadiversity measures, Chao1, Shannon, Simpson, and InvSimpson for bacteriaand fungi. The x-axis indicated the location the trees were replanted in, the previous tree stationor the corresponding alleyway position between the rows. The colour indicates the rootstock genotype identity. **Figure S3.** Alphadiversity measures, Chao1, Shannon, Simpson, and InvSimpson for bacteriaand fungi. The x-axis indicates the ARD score associated with each genotype. The shape indicates the planting location of the tree, the previous tree stationor the corresponding alleyway position between the rows.

## Data Availability

The datasets generated during and/or analysed during the current study are available in the NCBI database (Project PRJEB52534) https://www.ncbi.nlm.nih.gov/bioproject/PRJEB52534.

## Abbreviations

ARD: Apple replant disease
AMF: Arbuscular mycorrhizal fungi
PGPR: Plant growth promoting rhizobacteria
OTU: Operational taxonomic unit
NMDS: Non-metric multidimensional scaling
ADONIS: Multivariate analysis of variance using F-tests based on the sequential sum of squares
