# Supplementary material for: Inter-row cropping and rootstock genotype selection in a UK cider orchard to combat apple replant disease
Source: Phytopathol Res. 2023 Jul 4;5(1):28. doi: 10.1186/s42483-023-00184-y (PMC11116257; doi:10.1186/s42483-023-00184-y)
Supplement: Supplementary file 1 — Additional file 1: Table S1. ANOVA of log transformed girth data for planting position in alley or tree station and rootstock genotype. Block, Genotype, Year, and Position were set as fixed effects, whereas the tree pairwas treated as a random effect. Table S2. Summary of microbiome sequencing data. Table S3. Permutational multivariate analysis of variancebased on 1000 permutations of Bray–Curtis beta diversity accounted for by Block, Location, rootstock Genotype, ARD severity score, and interaction terms. [file 42483_2023_184_MOESM1_ESM.docx]

| **Table S1** ANOVA of log transformed girth data for planting position in alley or tree station and rootstock genotype. Block, Genotype, Year, and Position were set as fixed effects, whereas the tree pair (i.e. pair location within the orchard) was treated as a random effect | | | |
| --- | --- | --- | --- |
|  | **df** | **Sum Sq** | ***P* Value** |
| **Block** | 2 | 0.951 | 0.01 |
| **Genotype** | 6 | 4.740 | 3e-4 |
| **Year** | 5 | 36.34 | < 2e-16 |
| **Position** | 1 | 0.18 | 0.001 |
| **Position:Genotype** | 7 | 0.56 | 1.82e-5 |
| **Residuals** | 213 | 3.26 |  |

**Additional file 1**

| **Table S2** Summary of microbiome sequencing data | | | | | | | | |
| --- | --- | --- | --- | --- | --- | --- | --- | --- |
|  | **Total counts** | **Total OTUs** | **Number of OTU per sample** | | **Number of reads per sample** | | **Number of reads per OTU** | |
|  |  |  | Min | Max | Min | Max | Min | Max |
| *Bacteria* | 1549832 | 10883 | 1807 | 2445 | 25305 | 45970 | 2 | 2176 |
| *Fungi* | 4730414 | 4802 | 106 | 4474 | 2801 | 924636 | 2 | 49624 |

| **Table S3** Permutational Multivariate Analysis of Variance (ADONIS) based on 1000 permutations of Bray-Curtis beta diversity accounted for by Block, Location (Alleyway vs Tree Station), rootstock Genotype, ARD severity score, and interaction terms | | | | |
| --- | --- | --- | --- | --- |
|  |  | **df** | **Sum sq** | ***P* value** |
| *Bacteria* |  |  |  |  |
| Block |  | 2 | 0.21 | 9.9e-4 |
| Location |  | 1 | 0.13 | 9.9e-4 |
| Genotype |  | 6 | 0.25 | 0.56 |
|  | [1] ARD | 2 | 0.10 | 0.17 |
|  | [2] Between genotypes within ARD class | 4 | 0.15 |  |
| Location : Genotype |  | 6 | 0.21 | 0.91 |
|  | Location : [1] | 2 | 0.08 | 0.60 |
|  | Location : [2] | 4 | 0.13 |  |
| *Fungi* |  |  |  |  |
| Block |  | 2 | 1.81 | 9.9e-4 |
| Location |  | 1 | 0.48 | 8e-3 |
| Genotype |  | 6 | 1.29 | 0.64 |
|  | [1] ARD | 2 | 0.62 | 0.03 |
|  | [2] Between genotypes within ARD class | 4 | 0.67 |  |
| Location : Genotype |  | 6 | 1.37 | 0.48 |
|  | Location : [1] | 2 | 0.46 | 0.35 |
|  | Location : [2] | 4 | 0.91 |  |
