# Supplementary material for: Inter-row cropping and rootstock genotype selection in a UK cider orchard to combat apple replant disease
Source: Phytopathol Res. 2023 Jul 4;5(1):28. doi: 10.1186/s42483-023-00184-y (PMC11116257; doi:10.1186/s42483-023-00184-y)
Supplement: Supplementary file 2 — Additional file 2: Figure S1. Mean number of fruit counted on each tree in 2021. The shape of the point indicates the location in which the tree was planted. ● = Alleyway between previous rows, ▲ = Previous tree station. Figure S2. Alphadiversity measures, Chao1, Shannon, Simpson, and InvSimpson for bacteriaand fungi. The x-axis indicated the location the trees were replanted in, the previous tree stationor the corresponding alleyway position between the rows. The colour indicates the rootstock genotype identity. Figure S3. Alphadiversity measures, Chao1, Shannon, Simpson, and InvSimpson for bacteriaand fungi. The x-axis indicates the ARD score associated with each genotype. The shape indicates the planting location of the tree, the previous tree stationor the corresponding alleyway position between the rows. [file 42483_2023_184_MOESM2_ESM.docx]

**Additional file 2**


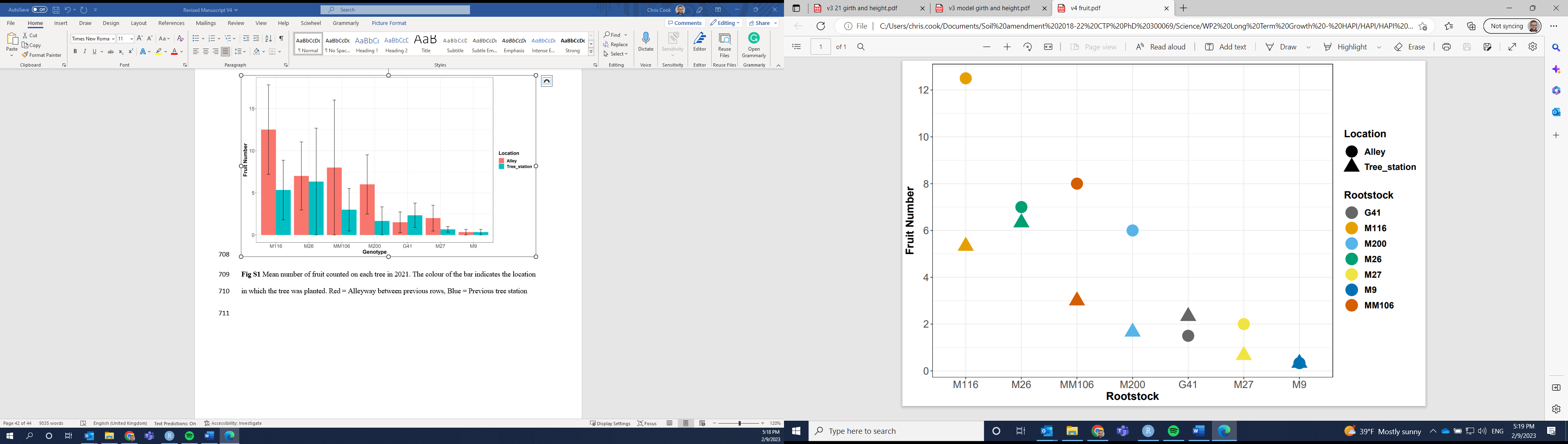


**Figure S1** Mean number of fruit counted on each tree in 2021. The shape of the point indicates the location in which the tree was planted. ● = Alleyway between previous rows, ▲ = Previous tree station


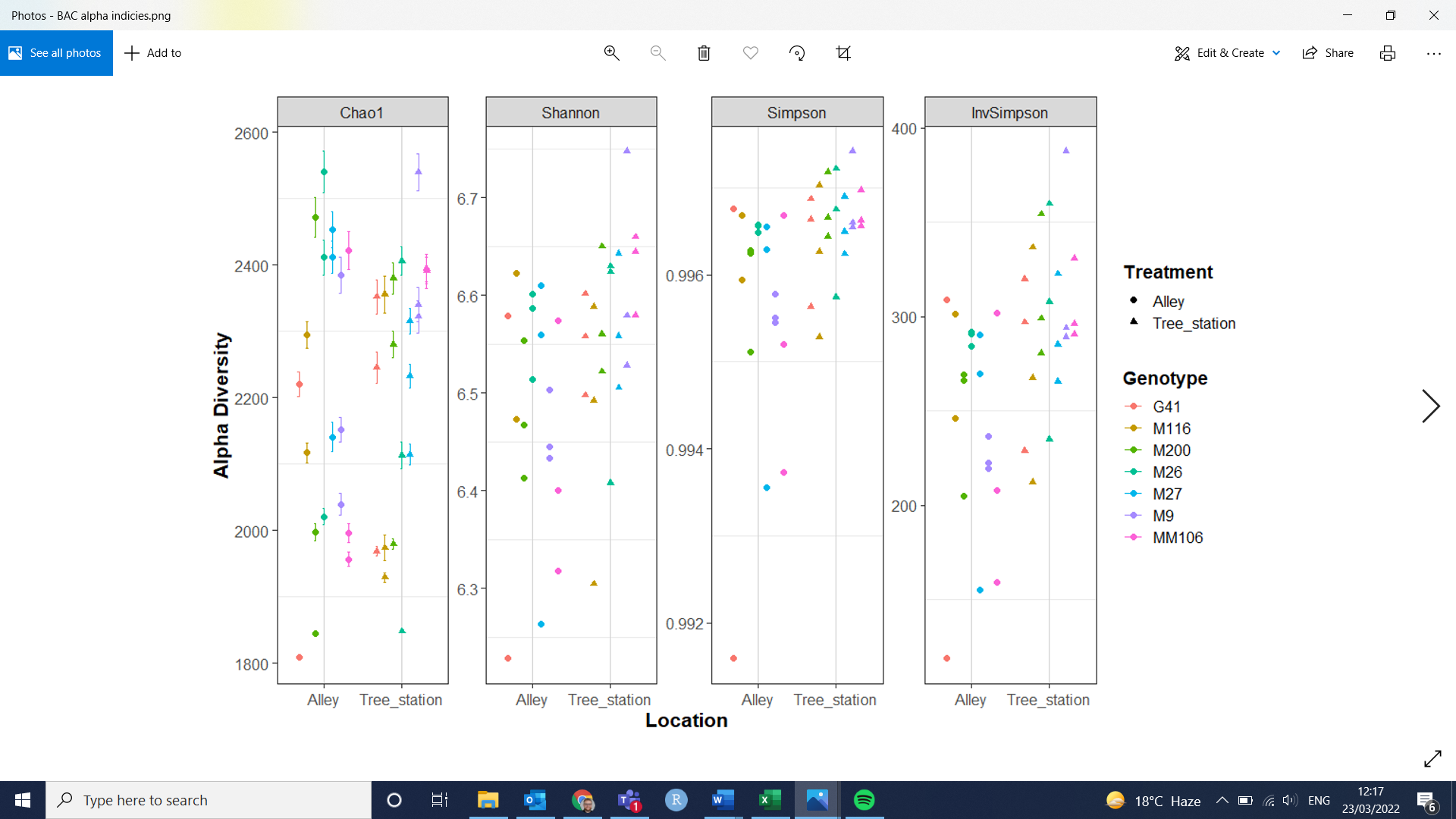

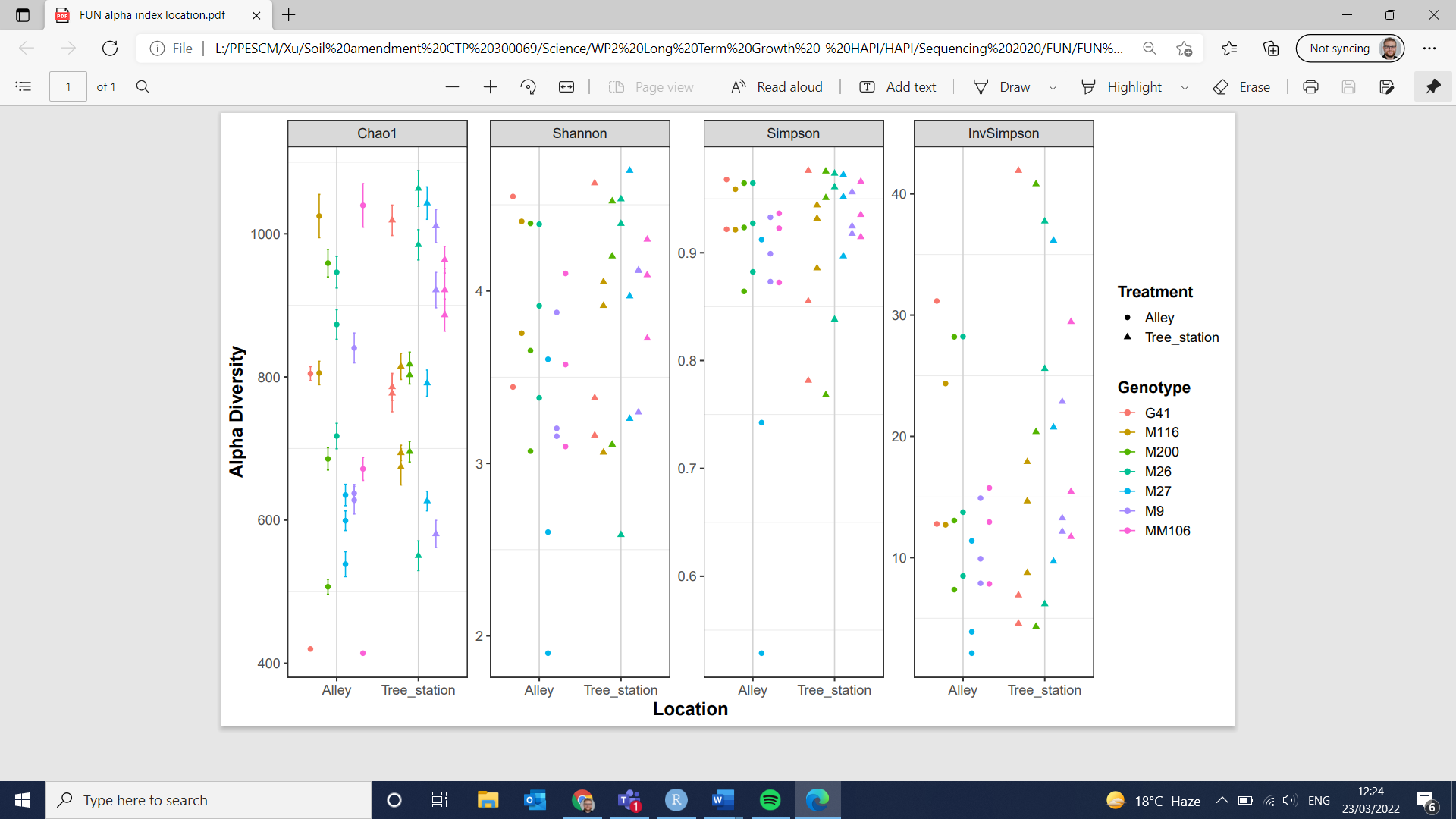


**a**

**b**

**Figure S2** Alpha (α) diversity measures, Chao1, Shannon, Simpson, and InvSimpson for bacteria (**a**) and fungi (**b**). The x-axis indicated the location the trees were replanted in, the previous tree station (▲) or the corresponding alleyway position between the rows (●). The colour indicates the rootstock genotype identity

**Figure S3** Alpha (α) diversity measures, Chao1, Shannon, Simpson, and InvSimpson for bacteria (**a**) and fungi (**b**). The x-axis indicates the ARD score associated with each genotype. The shape indicates the planting location of the tree, the previous tree station (▲) or the corresponding alleyway position between the rows (●)


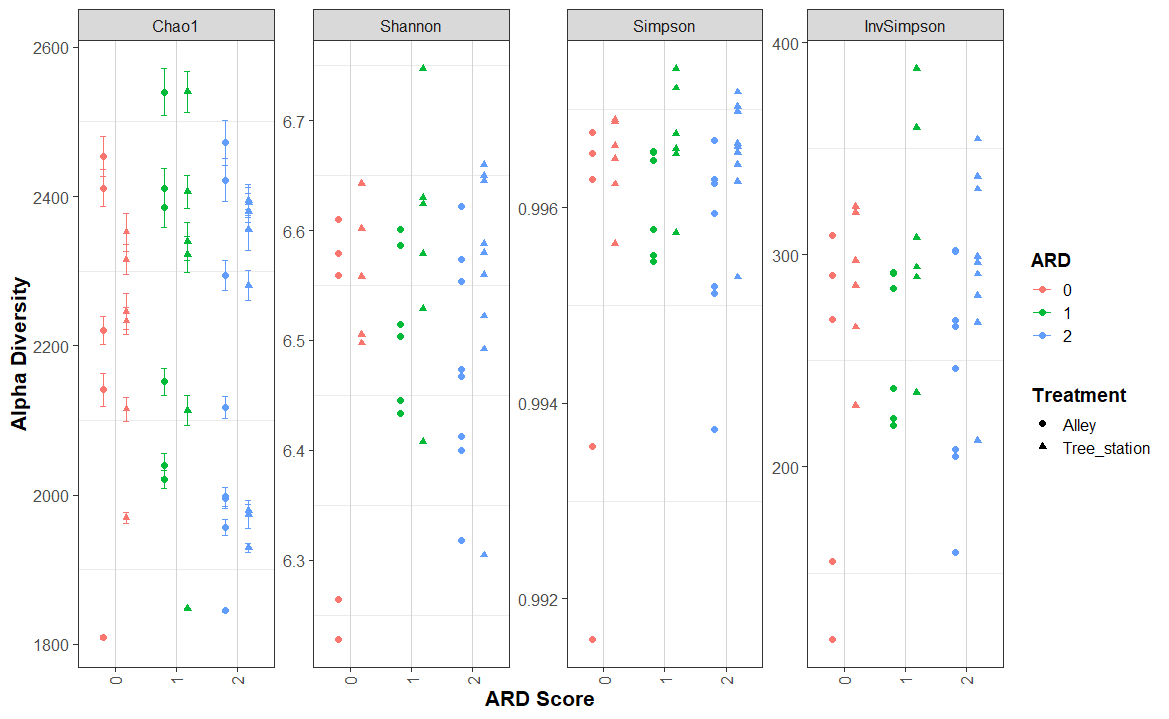

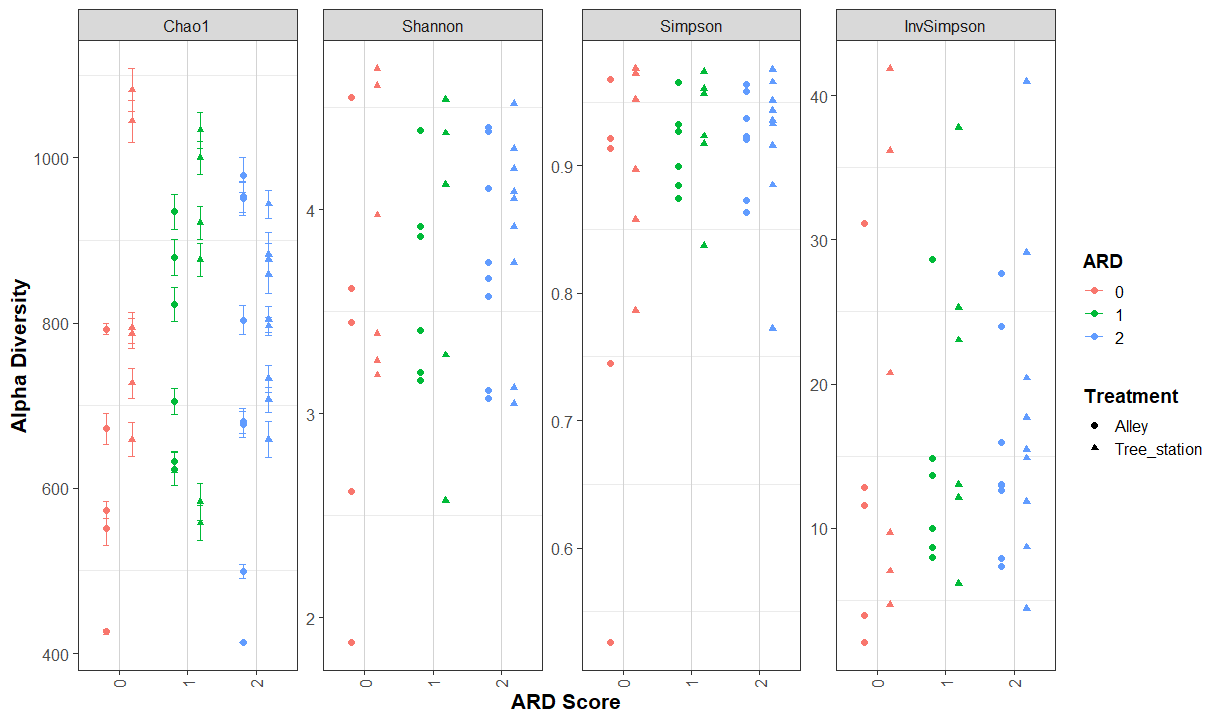


**a**

**b**
